# Metabolic Regulation Mechanism of *Ilex verticillata* in Response to Drought Stress: “First Inhibition Then Promotion” of Lipids and the Key Mediation of Glycerophospholipid Metabolic Pathway

**DOI:** 10.3390/biology14121673

**Published:** 2025-11-25

**Authors:** Yilin Xue, Yutang Cheng, Qiqi Li, Enhui Xing, Daoliang Yan

**Affiliations:** State Key Laboratory for Development and Utilization of Forest Food Resources, Zhejiang A&F University, Hangzhou 311300, China; xyl663627@163.com (Y.X.); 15184415178@163.com (Y.C.); lqq23401228@163.com (Q.L.); xingenhui16@163.com (E.X.)

**Keywords:** *Ilex verticillata*, drought stress, metabolomics

## Abstract

Background: *Ilex verticillata* belongs to the genus *Ilex* of the family Aquifoliaceae. It has extremely high ornamental value, and its drought tolerance is crucial for expanding its cultivation range. Therefore, studying the metabolic regulation mechanism of *I. verticillata* in response to drought stress at different stages under natural drought conditions provides a basis for the expanded cultivation of *I. verticillata* and important theoretical support for the drought-resistant breeding of woody plants. Methods: Two-year-old cutting seedlings (0.3~0.4 m high) of *I. verticillata* were used as experimental materials. A control group (normal watering) and a drought treatment group (natural drought after watering by the basin immersion method) were set up. Leaves were collected at the non-drought stage, and after 10, 20, and 30 days of drought and 1 day of rehydration for metabolomic analysis. Results: *I. verticillata* showed an obvious gradient of physiological responses under drought stress: the relative water content and chlorophyll content of leaves decreased gradually from the normal level and increased gradually after rehydration; the activity of antioxidant enzymes and the content of malondialdehyde increased continuously with the extension of drought stress and decreased significantly after rehydration. Metabolomic analysis revealed that the content of most lipid metabolites was down-regulated in the early stage of drought, which might reduce peroxidative damage by decreasing lipid synthesis; all lipid metabolites were up-regulated in the late stage of drought to alleviate stress by enhancing membrane stability; most lipid metabolites remained up-regulated after rehydration, promoting membrane structure reconstruction. Conclusions: The lipid metabolism of *I. verticillata* under drought stress presents a typical “first inhibition then promotion” pattern. Glycerophospholipid metabolism is a common metabolic pathway under both drought and rehydration conditions, which proves that this metabolic pathway is closely related to the drought-resistant characteristics of *I. verticillata*.

## Simple Summary

As an important ornamental and ecological tree species, *Ilex verticillata* relies heavily on its drought tolerance to expand its cultivation range. Under drought stress, plants maintain their survival through dynamic changes in physiological indicators and metabolic reprogramming, among which lipids and lipid-like metabolites play a key role in maintaining cell membrane stability and regulating membrane functions. Based on a drought stress experiment, this study determined physiological indicators including leaf relative water content, chlorophyll content, antioxidant enzyme activity, and malondialdehyde (MDA) content. Combined with untargeted metabolomics technology, it analyzed the dynamic variation patterns of lipids and lipid-like metabolites under drought and rehydration conditions, explored the coordinated changes between physiological indicators and lipid metabolites during the drought and rehydration processes of *I. verticillata*, and revealed its drought resistance mechanism. This research provides a theoretical basis for the breeding of drought-tolerant *I. verticillata* varieties.

## 1. Introduction

*Ilex verticillata* is a perennial deciduous shrub of the genus *Ilex* (family Aquifoliaceae), indigenous to Europe and North America [1]. Characterized by winter deciduousness, bright red fruits, and an extended fruit-retention period, this species exhibits high ornamental and commercial value, thereby occupying a prominent position in landscape greening projects and the cut-branch ornamental market [2,3]. The introduction, popularization and application of *I. verticillata* in China have promoted the development of its basic theoretical and applied research. Significant advancements have been made in various areas including tissue culture [4,5], stress resistance [6,7], transcriptome analysis [8], and in understanding fruit quality, growth, and development [9,10,11], with transcriptome analysis playing a pivotal role in revealing gene expression patterns and aiding in the improvement of crop varieties.

As one of the primary limiting factors constraining plant growth and geographical distribution [12], drought stress induces a series of physiological perturbations: it initially causes stomatal closure and a subsequent decline in photosynthetic efficiency, which further leads to osmotic stress and membrane lipid peroxidation, and ultimately results in the degradation of nucleic acids and proteins [13]. Plants adapt to drought environments through the coordinated regulation of multiple strategies, such as morphological structural modifications, physiological metabolic adjustments, and differential gene expression [14,15,16]. While extensive investigations into these adaptive mechanisms have been conducted in crops and forest tree species, research addressing the drought tolerance and its underlying mechanisms in *I. verticillata* remains relatively scarce.

Metabolomics, a discipline utilizing high-throughput detection technologies, conducts qualitative and quantitative analyses of all low-molecular-weight metabolites in biological samples from various growth stages or environmental conditions. Its primary objective is to identify key metabolites that play a role in specific biological processes, such as plant responses to adverse environmental stresses [17]. This approach has been widely applied to dissect how plants react to different environmental pressures, including drought, high temperatures, salinity, and pests [18,19,20]. For instance, in a metabolomic analysis of garlic (*Allium sativum*) seedlings under drought stress, differential metabolites were primarily enriched in the biosynthetic pathways of flavonoids and flavanones, among which caffeic acid and flavone O-malonylhexoside exhibited the most significant upregulation [21]. Through metabolomic profiling, Long et al. elucidated the dynamic variation patterns of metabolites during kernel development in oil-tea camellia (*Camellia oleifera*), revealing marked differences in metabolite composition across different developmental stages [22]. In a related study, Guo et al. [23] utilized comparative transcriptomics, metabolomics, and hormone analysis to elucidate the molecular mechanisms underlying maize (*Zea mays* B73) adaptation to combined drought and cold stresses, focusing on the stress treatment and recovery phases. They found that the combined stress induced transcriptome-related metabolomic changes, among which raffinose, trehalose-6-phosphate, and proline accumulated, and the abundance of monosaccharides increased.

Significant progress has been made in elucidating the physiological and molecular mechanisms that enable plants to respond to drought stress, yet there remains considerable variation in drought resistance strategies across different species, a reflection of their genetic diversity. As a species that prefers moisture, *I. verticillata* has rarely been studied in terms of its drought tolerance traits and associated response mechanisms. Therefore, the present study investigates the variation trends of drought-responsive differential metabolites and their related metabolic pathways in *I. verticillata*. By doing so, we aim to identify key targets and metabolic pathways involved in drought resistance metabolism, which may provide a theoretical basis for future breeding programs focused on developing drought-tolerant *I. verticillata* cultivars.

## 2. Materials and Methods

### 2.1. Experimental Materials and Treatments

Two-year-old rooted cuttings of *Ilex verticillata* ‘Oosterwijk’ were used as experimental materials. Seedlings with uniform growth were selected and transplanted into plastic pots with a diameter of 30 cm and a height of 40 cm. The cultivation medium was a mixture of peat soil and river sand at a volume ratio of 3:1. The seedlings were first acclimated for 30 days under regular management conditions: the temperature was controlled at 25–33 °C, the relative humidity at 80%, and the photoperiod was set to 12 h (day)/12 h (night). After acclimation, the experimental seedlings were randomly divided into 2 groups, with 50 seedlings in each group. Both groups were watered using the bottom irrigation method to ensure adequate water absorption. Subsequently, the drought group was subjected to natural drought stress (until the soil moisture drops to 20–30% of the field capacity), while the control group was watered normally throughout the experiment (the soil moisture was stably maintained at 70–80% of the field capacity). Leaf samples were collected from the plants on day 0, day 10, day 20, day 30 of drought treatment, and 1 day after rewatering (denoted as f1). The selection of these time points is mainly based on three considerations: first, it aligns with the stage-specific characteristics of drought stress, including short-term inhibition, mid-term transition, and long-term adaptation; second, it matches the time scale of key processes in lipid metabolism; third, it balances data integrity with experimental practicality. Each sampling was conducted between 9:00 and 10:00 a.m. The collected leaves were immediately quick-frozen in liquid nitrogen and then stored in a −80 °C refrigerator for subsequent analysis. Leaves from 3 *I. verticillata* seedlings were mixed to form one biological replicate, and a total of 3 biological replicates were set up in the experiment.

### 2.2. Determination of Physiological Indicators

#### 2.2.1. Determination of Chlorophyll Content

Leaves of *Ilex verticillata* were sampled using a punch with a pore size of 6.78 mm. Subsequently, 10 mL of 95% ethanol solution was added, and the leaves were soaked in the dark for 45 h until they completely faded. The absorbance values at wavelengths of 470 nm, 645 nm, and 663 nm were measured, and the contents of chlorophyll a, chlorophyll b, and total chlorophyll per gram of leaves were calculated according to the Amon equation:

Chlorophyll a (mg/g fresh weight) = (12.7D_663_ − 2.69D_645_) × [V/(1000 × Wf)]

Chlorophyll b (mg/g fresh weight) = (22.9D_645_ − 4.63D_663_) × [V/(1000 × Wf)]

Total chlorophyll content (mg/g fresh weight) = (20.2D_645_ + 8.2D_663_) × [V/(1000 × Wf)]

In the formulas:

D = optical density reading;

V = total volume of chlorophyll extract (10 mL);

Wf = fresh weight of the leaves used (g).

#### 2.2.2. Determination of Leaf Relative Water Content (RWC)

First, measure the fresh weight of the leaf (denoted as Wf). Then, immerse the leaf in distilled water for approximately 48 h until it reaches a state of full water absorption. Take out the leaf, use absorbent paper to remove surface moisture, and quickly place it in a pre-weighed container for weighing. After that, immerse the leaf in distilled water again, take it out, wipe off the external moisture, and weigh it again. Repeat this process until the weight becomes stable. At this point, the weight is the saturated weight of the leaf after water absorption (denoted as Wt). Subsequently, dry the sample and measure the dry weight of the leaf (denoted as Wd). The relative water content is calculated using the formula:

Relative Water Content = [(Wf − Wd)/(Wt − Wd)] × 100%

#### 2.2.3. Determination of Malondialdehyde Content

Thiobarbituric Acid Method: Take a clean test tube, add 1 mL of enzyme extract, then add 3 mL of 10% trichloroacetic acid (TCA) and 1 mL of 0.6% thiobarbituric acid (TBA), shake well. Incubate the mixture in a 95 °C water bath for 30 min, cool it rapidly, then centrifuge at 4000 rpm for 10 min. Take the supernatant and measure the absorbance at wavelengths of 600 nm, 532 nm, and 450 nm, respectively. The control is 0.6% TBA. Calculation method:

MDA content (μmol/g) = [6.452 × (A_532_ − A_600_) − 0.559 × A_450_] × V1/(W × V2)

where V1—Total volume of the extract (mL); V2—Volume of the extract used for determination (mL); W—Fresh weight of the sample.

#### 2.2.4. Determination of Antioxidant Enzyme Activity

For the determination, 0.1 g of *Ilex verticillata* leaves was taken and placed in a pre-cooled mortar. Then, 2 mL of pre-cooled 50 mmol·L^−1^ phosphate buffer (pH 7.8) was added to grind the leaves into a slurry. The mortar was rinsed with additional phosphate buffer, and the final volume of the mixture was adjusted to 8 mL. The mixture was centrifuged at 10,000 rpm for 15 min at 4 °C, and the supernatant was used as the enzyme extract. The enzyme extract was stored at 0–4 °C for later use to determine the activities of superoxide dismutase (SOD) and peroxidase (POD).

(1) Determination of POD activity: The guaiacol method was used to determine POD activity. When preparing the reaction solution, 100 mL of 0.05 mol·L^−1^ phosphate buffer (pH 6.0), 100 mL of RO water, 100 μL of guaiacol, and 100 μL of 30% hydrogen peroxide were sequentially added to a beaker. After thorough mixing, the solution was transferred to a reagent bottle. First, 0.1 mL of the enzyme extract was injected into a cuvette, followed by the addition of 3 mL of the POD reaction reagent. A reaction system without enzyme extract was used as the blank control, and the absorbance was quickly measured at a wavelength of 470 nm. Measurements were taken at 15-s intervals, and data were recorded continuously for the first 3 min. Three parallel samples were set up for each group of experiments, and the final result was the arithmetic mean of three measurements. The calculation formula is as follows:
  POD activity (U·g^−1^·min^−1^) = (ΔA_470_ × Vt)/(0.1 × Wf × Vs × t)
  In the formula:
  Wf = fresh weight of the sample;
  ΔA_470_ = change in absorbance during the reaction time (average change value);
  Vt = total volume of the enzyme extract;
  Vs = volume of the enzyme extract used for determination;
  t = reaction time.
(2) Determination of SOD Activity: The activity of superoxide dismutase (SOD) was determined according to the method described by Beauchamp and Fridovich, with the calculation formula as follows:
  SOD Activity = [(Ao − As) × Vt]/(0.5 × Ao × Wf × Vi)
  In the formula:
  Ao = light absorption value of the reference control tube;
  As = light absorption value of the sample tube;
  Vt = total volume of the sample solution (mL);
  Vi = volume of the sample used for determination (mL);
  Wf = fresh weight of the sample (g).
(3) Determination of CAT Activity: The activity of catalase (CAT) was measured using a catalase (CAT) assay kit, which was purchased from Suzhou Geruisi Biotechnology Co., Ltd. (Suzhou, China).

### 2.3. Metabolomics Analysis Methods

#### 2.3.1. Sample Collection

Weigh 50 mg of sample into a 2.0 mL EP tube, add 500 μL of 80% ice-cold methanol solution, and put a small amount of steel beads into the tube to crush the sample using a grinder. Place the tube in a −20 °C refrigerator and let it stand for 30 min to precipitate proteins in the sample. After centrifugation at 20,000 rcf for 15 min, transfer 400 μL of the supernatant to another EP tube. Freeze-dry the supernatant, then add 100 μL of 50% ice-cold methanol solution to redissolve it. Perform another centrifugation at 20,000 rcf for 15 min, and then transfer the supernatant to a sample vial for UPLC-HRMS detection. For each sample, take 15 μL of the extract in equal amounts and mix them to form a QC sample, which is also used for UPLC-HRMS detection. All operations are carried out on ice throughout the process.

#### 2.3.2. Chromatographic Conditions

The acquisition of all samples was conducted using the LC-MS system, adhering to the established machine orders, a process that is standard in the field for ensuring accurate and reliable data. Firstly, all chromatographic separations were performed using an UltiMate 3000 UPLC System (Thermo Fisher Scientific, Bremen, Germany). The separation was performed using a ACQUITY UPLC HSS T3 column (Waters Corporation, Milford, MA, USA), featuring a 1.8 µm particle size, 2.1 mm internal diameter, and 100 mm length, designed for efficient reversed phase chromatography. The column oven was maintained at 40 °C. Then, the mobile phase consisted of solvent A (5 mM ammonium acetate and 5 mM acetic acid) and solvent B (Acetonitrile). Flow rate was 0.3 mL/min and the mobile phase consisted of solvent A. Gradient elution conditions were set as follows: 0~0.8 min, 2% B; 0.8~2.8 min, 2% to 70% B; 2.8~5.6 min, 70% to 90% B; 5.6~6.4 min, 90% to 100% B; 6.4~8.0 min, 100% B; 8.0~8.1 min, 100% to 2% B; 8.1~10 min, 2% B.

#### 2.3.3. Mass Spectrometry Conditions

The high-resolution mass spectrometer used for data acquisition was the Q-Exactive Plus (Thermo Fisher Scientific, Bremen, Germany). Each sample was analyzed once in positive ion mode and once in negative ion mode. The shield gas pressure of the ion source was set to 0, the pressure of Gas 1 (auxiliary gas) was 10, and the pressure of Gas 2 (sheath gas) was 35. The ion source temperature was maintained at 350 °C. The voltage was +3500 V in positive ion mode and −3000 V in negative ion mode. Data acquisition was performed using the DDA (Data-Dependent Acquisition) mode. During the acquisition process, a QC (Quality Control) sample was scanned every 10 samples, and the mass deviation between QC samples was used to correct the systematic errors of the entire batch of experiments.

### 2.4. Statistical Analysis of Data

Statistical analysis was conducted using R (version 4.0.0). The raw protein intensity was normalized using the ‘medium’ method. Hierarchical clustering was performed using pheatmap package (version 1.0.12). Principal component analysis (PCA) was performed using metaX package (version 2.0.0). The PLSDA analysis is performed by the R package ropls (version 1.26.4) and the VIP values of each variable are calculated. Correlation analysis was performed using the Pearson correlation coefficient from the ‘cor’ package (version 4.1.2). The three conditions of *p* Value < 0.05, difference multiple > 1.2 obtained by *t*-test and VIP calculated by PLSDA analysis simultaneously met the screening of the final metabolites with significant differences. Individual protein sequences were annotated using hypergeometric-based enrichment analysis within the KEGG Pathway framework. The software GSEA (version 4.1.0) and MSigDB database were utilized for gene set enrichment analysis to assess the behavior of a specific set of genes within a KEGG pathway under different conditions. Meeting this condition |NES| > 1, NOM *p*-value < 0.05, FDR q-value < 0.25 were considered to be significantly different between the two groups. The network map is drawn according to the pathway where the metabolite is located.

## 3. Results and Analysis

### 3.1. Effects of Drought Stress on Chlorophyll Content in Leaves of Ilex verticillata

**Figure 1:**
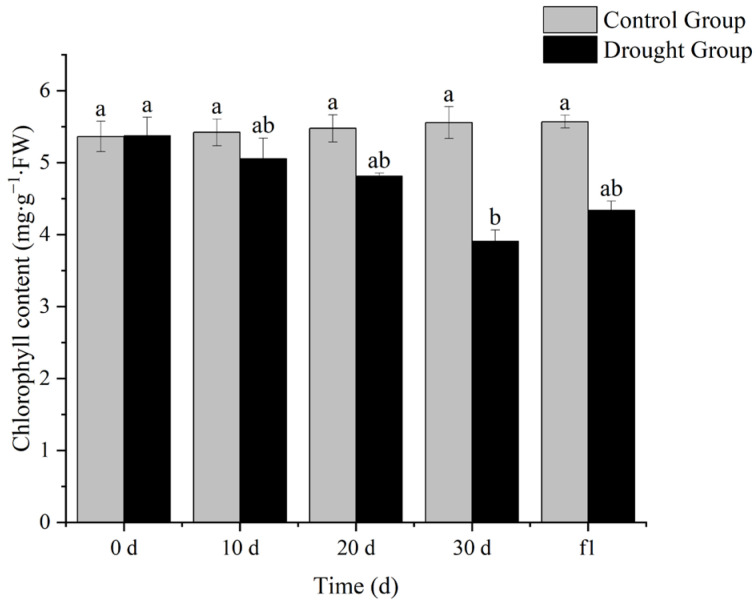
Effects of drought stress and rehydration on chlorophyll content in leaves of *I. verticillata*. Note: 0 d, 10 d, 20 d, and 30 d refer to drought treatment durations, while f1 denotes 1 day of rewatering after 30 d of drought; gray bars represent the Control Group and black bars represent the Drought Group; the ordinate is chlorophyll content with the unit of percentage (mg∙g^−1^∙FW); lowercase letters (a, b, ab) above the bars are significance markers: the same letter indicates no significant difference between groups (*p* > 0.05), while different letters indicate significant differences (*p* < 0.05).

Photosynthesis is the process by which plants convert CO_2_ and H_2_O into carbon-containing organic compounds, which supplies energy for plants and maintains their normal growth and development. Chlorophyll is a key pigment closely related to photosynthesis and plays a core role in the process of light absorption. As shown in Figure 1, the chlorophyll content in the leaves of *I. verticillata* showed a decreasing trend with the increase in drought duration, and there was a significant difference compared with the control group at the same period when drought stress lasted for 30 days. After rewatering treatment, the chlorophyll content increased to a certain extent. These results indicate that drought stress reduces the chlorophyll content in the leaves of *I. verticillata*, inhibits its photosynthesis, and affects the ability of *I. verticillata* to cope with drought stress.

### 3.2. Effects of Drought Stress on Relative Water Content in Leaves of Ilex verticillata

**Figure 2:**
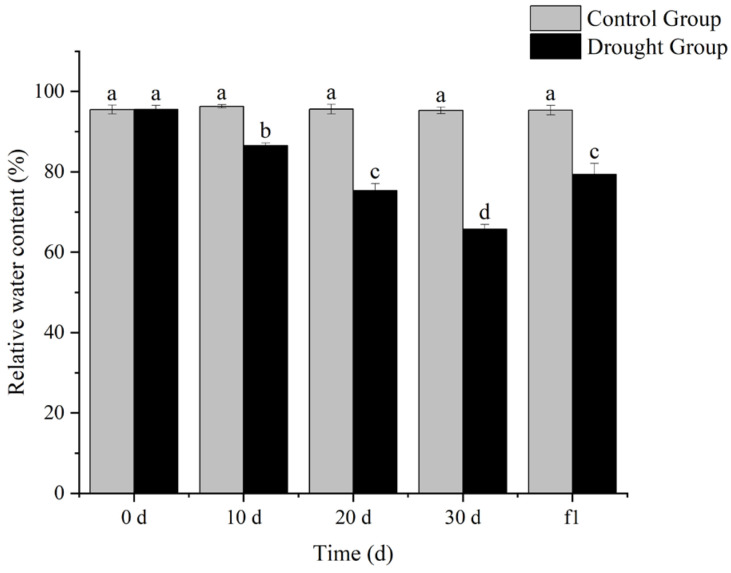
Effects of drought stress and rehydration on the relative water content of *I. verticillata* leaves. Note: 0 d, 10 d, 20 d, and 30 d refer to drought treatment durations, while f1 denotes 1 day of rewatering after 30 d of drought; gray bars represent the Control Group and black bars represent the Drought Group; the ordinate is relative water content with the unit of percentage (%); lowercase letters (a, b, c, d) above the bars are significance markers: the same letter indicates no significant difference between groups (*p* > 0.05), while different letters indicate significant differences (*p* < 0.05).

The relative water content (RWC) of plant leaves reflects the water status and water retention capacity of plants, and serves as a crucial indicator for evaluating the strength of plants’ drought resistance. As shown in Figure 2, there was no significant difference in the leaf RWC of *I. verticillata* between the treatment group and the control group on day 0 of treatment. With the extension of drought duration, the leaf RWC decreased continuously, and all values showed significant differences compared with the control group at the same period. After rewatering treatment, the leaf RWC of *I. verticillata* increased to a certain extent. These results indicate that drought stress reduces the water retention capacity of *I. verticillata* leaves, while rewatering can restore the plant’s water status to some degree. This suggests that *I. verticillata* possesses a certain adaptive mechanism when facing drought stress, enabling it to cope with drought environments by regulating leaf water content.

### 3.3. Effects of Drought Stress on Malondialdehyde (MDA) Content in Ilex verticillata

Under stress conditions, the content of superoxide free radicals in plants increases sharply, triggering lipid peroxidation and generating malondialdehyde (MDA). Excessive accumulation of this substance can promote the cross-linking and polymerization of biological macromolecules such as proteins and nucleic acids, thereby altering the structure and function of cell membranes.

**Figure 3:**
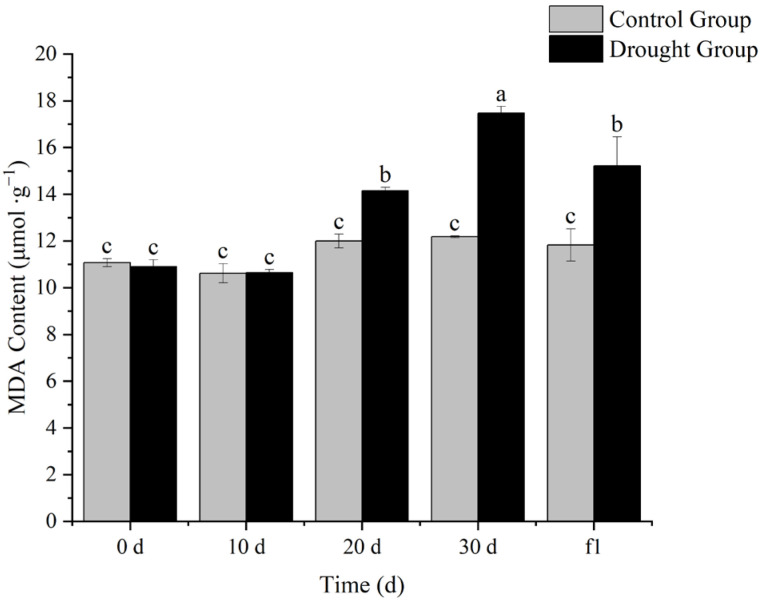
Effects of drought stress and rehydration treatment on malondialdehyde content of *I. verticillata.* Note: 0 d, 10 d, 20 d, and 30 d refer to drought treatment durations, while f1 denotes 1 day of rewatering after 30 d of drought; gray bars represent the Control Group and black bars represent the Drought Group; the ordinate is MDA content with the unit of percentage (μmol∙g^−1^); lowercase letters (a, b, c) above the bars are significance markers: the same letter indicates no significant difference between groups (*p* > 0.05), while different letters indicate significant differences (*p* < 0.05).

As shown in Figure 3, there was no significant difference between the treatment group and the control group on day 0 of treatment. On day 20 of drought stress, a significant difference was observed between the treatment group and the control group of the same period. The significant difference further increased on day 30 of drought stress, and was alleviated after rewatering. The differences in MDA content indicate that drought stress induces membrane lipid peroxidation in *I. verticillata*, which in turn affects its ability to resist drought.

### 3.4. Effects of Drought Stress on Antioxidant Enzyme Activities in Ilex verticillata

**Figure 4:**
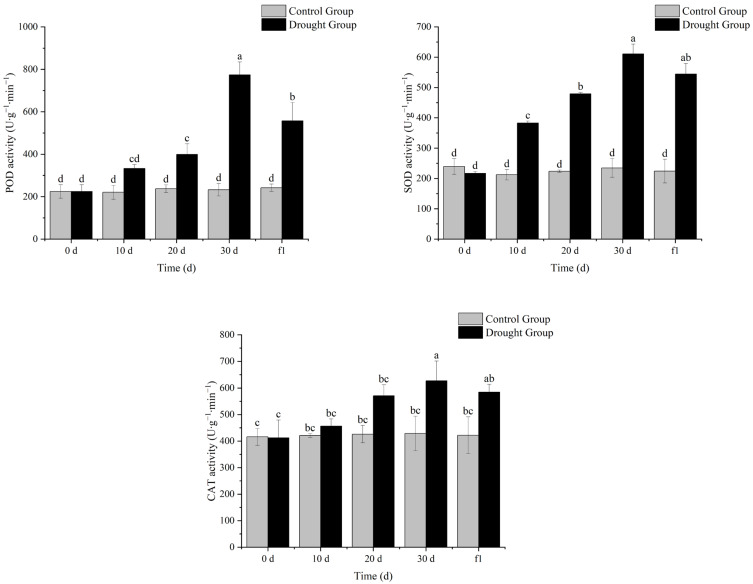
Effects of drought stress and rehydration treatment on the antioxidant enzyme content of *I. verticillata.* Note: 0 d, 10 d, 20 d, and 30 d refer to drought treatment durations, while f1 denotes 1 day of rewatering after 30 d of drought; gray bars represent the Control Group and black bars represent the Drought Group; the ordinate is the activity of each antioxidant enzyme with the unit of percentage (U∙g^−1^∙min^−1^); lowercase letters (a, b, c, d, ab, bc, cd) above the bars are significance markers: the same letter indicates no significant difference between groups (*p* > 0.05), while different letters indicate significant differences (*p* < 0.05).

As shown in Figure 4, with the extension of drought stress, the antioxidant enzyme activity of *I. verticillata* increased continuously, and decreased to some extent after rewatering. In particular, the activity of superoxide dismutase (SOD) showed a significant difference compared with the control group at the same period on the 10th day of drought, while the activities of peroxidase (POD) and catalase (CAT) showed significant differences compared with the control group at the same period on the 20th and 30th days of drought, respectively. These results indicate that under drought stress, membrane lipid peroxidation occurs in *I. verticillata*, and the plant resists drought by increasing antioxidant enzyme activity. The longer the drought stress lasts, the higher the antioxidant enzyme activity of *I. verticillata* becomes, which suggests that *I. verticillata* has a certain adaptive mechanism and can gradually enhance its antioxidant capacity to cope with more severe drought stress.

### 3.5. Sample Correlation Analysis

**Figure 5:**
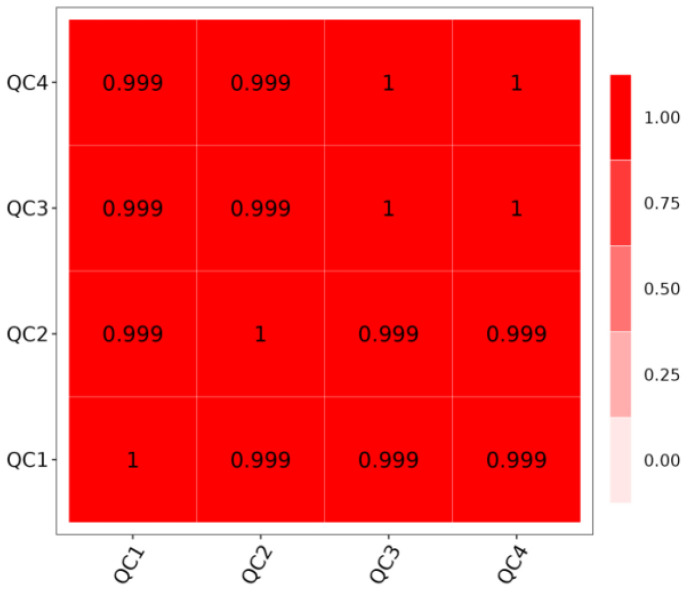
Correlation analysis of *I. verticillata*.

Quality Control (QC) is a crucial step to ensure the reliability and accuracy of experimental results. As demonstrated in Figure 5, the sample correlation coefficients are all near 1, signifying strong linear relationships and high repeatability among the samples. This high repeatability is indicative of the samples’ suitability for further analysis.

### 3.6. PCA of Total Samples

**Figure 6:**
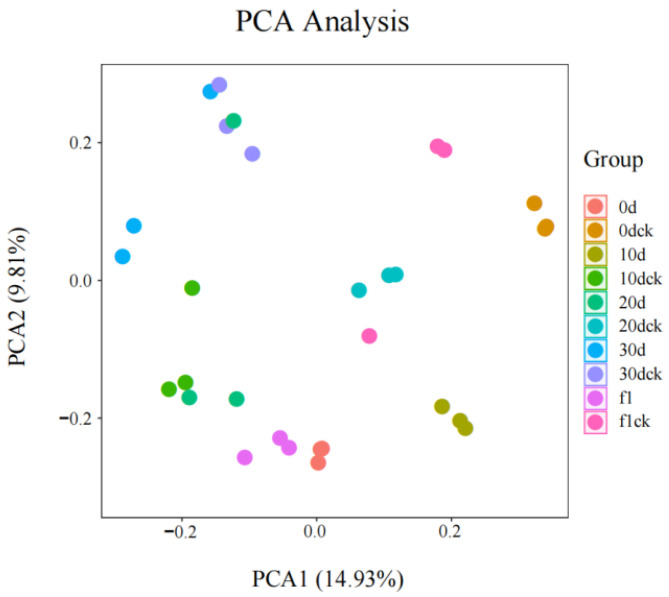
Principal Component Analysis (PCA) of *I. verticillata*.

Principal Component Analysis (PCA) was employed to evaluate the overall distribution among all tested samples and the reliability of the analysis process, with the variance explained by the first principal component (PC1) being 14.93%, indicating a clear distinguishability between groups and good aggregation within groups. As shown in Figure 6, the 10 treatment groups of samples exhibited clear distinguishability between groups, while the samples within each group showed good aggregation and repeatability, indicating that the tested samples were reliable. The PCA results revealed that the direction with the largest total data variation (PC1) explained 14.93% of the total data variability, and the direction with the second-largest total data variation (PC2) explained 9.81% of the total data variability. The above results demonstrated that there were significant differences in the metabolites among the 10 groups of samples.

### 3.7. Differential Metabolite Analysis

Differential metabolites were screened using the thresholds of VIP > 1.0, FC > 2.0 or FC < 0.5, and *p* value < 0.05, with the results shown in Table 1. Analysis of Table 1 reveals the following:

After 10 days of drought stress, the study identified 68 metabolites with differential expression, comprising 40 metabolites that were significantly up-regulated and 28 that were significantly down-regulated. After 20 days of drought stress, a comparable number of metabolites were up-regulated and down-regulated, with 113 metabolites showing increased expression and 117 metabolites showing decreased expression. On the 30th day of drought stress, the number of differential metabolites decreased to 165, and the number of up-regulated metabolites was significantly greater than that of down-regulated metabolites; On the 1st day after rewatering, the number of differential metabolites reached the maximum (239), including 130 up-regulated metabolites and 109 down-regulated metabolites.

### 3.8. KEGG Enrichment Analysis of Differential Metabolites

KEGG enrichment analysis of differential metabolites showed that:

On the 10th day of drought stress, differential metabolites were mostly enriched in the “biosynthesis of secondary metabolites” pathway. On the 20th day of drought stress, they were mostly enriched in “metabolic pathways”. On the 30th day of drought stress, they were enriched in “glycerophospholipid metabolism”. On the 1st day after rewatering, they were again mostly enriched in “metabolic pathways”.

**Figure 7:**
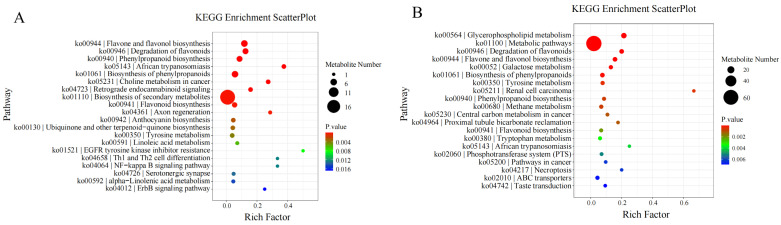

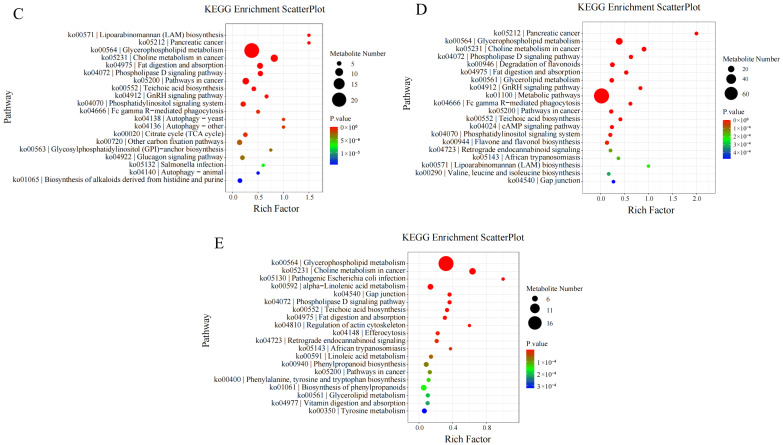
Bubble plot of KEGG enrichment analysis of differential metabolites in drought treatment. Note: KEGG enrichment analyses: (**A**): 10 d vs. 10 dck; (**B**): 20 d vs. 20 dck; (**C**): 30 d vs. 30 dck; (**D**): f1 vs. f1 ck; (**E**): 0 dck vs. 10 d vs. 20 d vs. 30 d vs. f1.

Furthermore, longitudinal KEGG enrichment analysis of differential metabolites involved in *I. verticillata*’s response to drought stress revealed that throughout the entire drought and rewatering process, differential metabolites were mainly enriched in the “glycerophospholipid metabolism” pathway (Figure 7).

### 3.9. Cluster Analysis of Differential Metabolites

**Figure 8:**
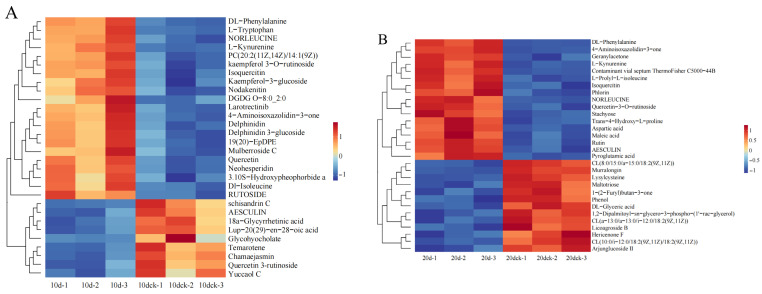

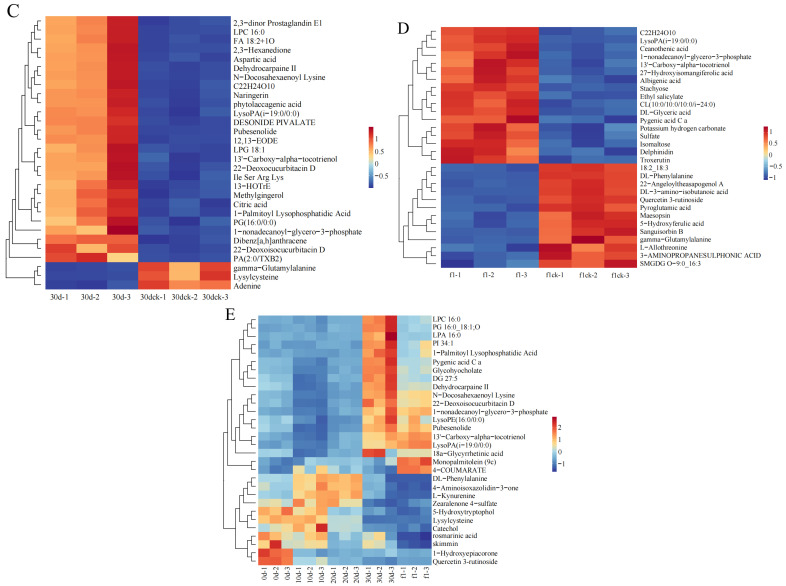
Hierarchical clustering heatmap of differential metabolites in *I. verticillata*. Note: Heatmaps of cluster analysis for top 30 differential metabolites: (**A**): 10 d vs. 10 dck; (**B**): 20 d vs. 20 dck; (**C**): 30 d vs. 30 dck; (**D**): f1 vs. f1 ck; (**E**): Among 10 d, 20 d, 30 d, and f1.

The heatmap of cluster analysis on the top 30 differential metabolites in each group indicates that *I. verticillata* exhibits varying metabolite profiles across different drought periods, as observed in Figure 8.

On the 10th day of drought stress, most of the differentially expressed metabolites were phenylpropanoids and polyketides enriched in the biosynthetic pathway of secondary metabolites. Among them, all were up-regulated except for aesculin, schisandrin C, and yuccaol C, whose expression levels were down-regulated. On the 20th day of drought stress, the differentially expressed metabolites were mostly organic acids and their derivatives, including maleic acid, pyroglutamic acid, and aspartic acid. On the 30th day of drought stress, the differentially expressed metabolites were mostly lipids and lipid-like molecules enriched in the glycerophospholipid metabolism pathway, such as 1-acyl-sn-glycero-3-phosphoglycerol (LPG 18:1), phosphatidylglycerol, and phosphatidic acid. On the 1st day after rewatering, the differentially expressed metabolites were mostly enriched in the glycerophospholipid metabolism pathway and metabolic pathways. Among them, lipid and lipid-like molecules accounted for the largest proportion of metabolites, and the expression levels of most of these metabolites enriched in the glycerophospholipid metabolism pathway (e.g., lysophosphatidic acid, cardiolipin were up-regulated.

In addition, the screening of longitudinally differentially expressed metabolites during the drought and rewatering process showed that these metabolites were mainly enriched in the glycerophospholipid metabolism pathway and metabolic pathways. Most of these metabolites (e.g., 1-acyl-sn-glycerol-3-phosphate, Phosphatidylglycerol, 1-phosphatidyl-D-inositol) were up-regulated with the extension of drought stress duration and down-regulated after rewatering.

### 3.10. Analysis of Enriched Pathways for Differential Metabolites

Analysis of the top 20 KEGG metabolic pathways revealed that drought stress impacted the glycerophospholipid metabolism and other metabolic pathways in *I. verticillata*. Based on these findings, we compared the differential metabolites related to the glycerophospholipid metabolic pathway between two pairs of groups: 30 d vs. 30 dck, and f1 vs. f1 ck. The results showed that: on the 30th day of drought stress, the content of phosphatidylcholine in the leaves of *I. verticillata* was lower than that in the control group (CK), while the contents of phosphatidic acid, cardiolipin, and lysophosphates were higher. After rewatering, the contents of phosphatidic acid and cardiolipin in the leaves of *I. verticillata* were lower than those in the control group, while the contents of phosphatidylglycerol, lecithin, and CDP-choline were higher than those in the control group.

These results suggest that under drought stress, *I. verticillata* may regulate the composition of membrane phospholipids to cope with water deficit and maintain the integrity of cell membranes. After rewatering, it may restore the normal function of membranes and promote hydration by increasing specific phospholipid components.

In addition, by comparing the differentially expressed metabolites related to metabolic pathways between the two stages (20 d vs. 20 dck and f1 vs. f1 ck), it was found that on the 20th day of drought stress, the contents of DL-phenylalanine and pyroglutamic acid were higher than those in the control group, while the contents of cardiolipin and DL-glyceric acid were lower than those in the control group.

Following rewatering, the regulation trends of the aforementioned metabolites reversed compared to those observed on the 20th day of drought stress. These findings suggest that, under drought stress, *I. verticillata* may mitigate water deficiency by up-regulating specific stress-resistant metabolites (e.g., phenylalanine and pyroglutamic acid), and simultaneously regulating membrane lipids and energy metabolism to minimize consumption. After rewatering, it restores normal growth and metabolic status by adjusting the levels of these metabolites.

## 4. Discussion

Relative water content, chlorophyll content, MDA content, and antioxidant enzyme activity are important physiological indicators when plants are subjected to drought stress. Determining these indicators helps quantify the intensity of drought stress and analyze the physiological mechanisms of plant drought resistance.

In this study, *Ilex verticillata* showed a clear gradient of physiological responses during drought stress: the relative water content of leaves continued to decrease with the extension of drought duration, with a significant difference from the control group at 30 days, and gradually increased after rehydration, indicating that the plants responded to stress by dynamically regulating the balance between water absorption and transpiration. The decreasing trend of chlorophyll content is closely related to the protection of the photosynthetic system—water deficit inhibits the activity of chlorophyll synthase and accelerates chlorophyll degradation, thereby reducing light energy capture to decrease transpiration water consumption, which is a typical “drought avoidance” strategy. The continuous increase in antioxidant enzyme activity reflects the “active defense” mechanism, which reduces membrane lipid peroxidation by scavenging reactive oxygen species. However, the MDA content increased significantly after 20 days of drought, reflecting the progressive process of cell membrane structure damage with the aggravation of stress. The reverse adjustment of each indicator after rehydration confirms the reversibility and repair potential of the physiological responses of *I. verticillata*.

Untargeted metabolomics technology has the advantages of high throughput, integrity, and objectivity. Leveraging multivariate statistical analysis techniques, the study conducts both qualitative and quantitative assessments of the chemical compositions of plants across various treatments. This facilitates a comprehensive exploration of the characteristics and variation patterns of different chemical components during plant growth and development, leading to its widespread application in plant research. KEGG enrichment analysis, a method that involves data preprocessing, functional annotation, and pathway enrichment analysis, reveals the effects of different treatment groups on plants by comparing metabolome data with the KEGG database.

In this study, untargeted metabolomic analysis was performed on each treatment group under drought and rewatering treatments, with a focus on the comparison groups of 20 d vs. 20 dck, 30 d vs. 30 dck, and f1 vs. f1 ck. The analysis results showed that in the 30 d vs. 30 dck comparison group, the number of significantly up-regulated differential metabolites was greater than that of significantly down-regulated ones; while in the 20 d vs. 20 dck and f1 vs. f1 ck comparison groups, the total number of significantly up-regulated and down-regulated differential metabolites was basically the same. Based on the above results, the top 30 metabolites in the three comparison groups were selected for analysis. It was found that the three comparison groups contained relatively high contents of three types of metabolites, namely lipids and lipid-like molecules, phenylpropanoids and polyketides, and organic acids and their derivatives.

Lipids, including phospholipids and triglycerides, alongside lipid-like substances like carotenoids, are essential constituents of plant cell membranes. They play a pivotal role in preserving the structural integrity and ensuring the fluidity of these membranes, which is critical for cellular functions. In this study, among the top 30 metabolites, on the 20th day of drought stress, there were 5 lipid and lipid-like metabolites; on the 30th day, the number increased to 17; and on the 1st day after rewatering, there were 11 lipid and lipid-like metabolites. Among them, most lipid and lipid-like metabolite contents were down-regulated on the 20th day of drought stress. This may occur because drought increased reactive oxygen species (ROS) levels in *I. verticillata*, causing lipid peroxidation and cell membrane damage. Therefore, plants may reduce lipid synthesis to lower peroxide production, thereby alleviating cell membrane damage. On the 30th day of drought stress, the contents of all 17 lipid and lipid-li. The metabolites were up-regulated, indicating that drought stress can damage the cell membrane of *I. verticillata*. However, the accumulation of lipids and lipid-like substances can mitigate this issue by enhancing the stability and protective capacity of the cell membrane. Following rewatering treatment, it was observed that most lipid and lipid-like metabolites remained up-regulated, whereas a minority were down-regulated. This may be because rewatering *I. verticillata* may stimulate lipid synthesis and membrane reconstruction, thus increasing the levels of related lipid metabolites. It can be seen that the underlying mechanisms of the aforementioned physiological changes are closely related to the dynamic remodeling of lipid metabolism in metabolomics, and that lipid metabolism under drought stress in *I. verticillata* exhibits a typical ‘suppression followed by promotion’ pattern. Shen et al. [24] found that lipid metabolites in tea leaves exhibited an obvious temporal pattern of “first inhibition then promotion” under drought stress. Michael Ackah made the same observation in *Morus alba* L. [25].

In addition, Ling et al. [26] discovered that under drought stress, phosphatidic acid (PA) is the most critical responsive lipid in somatic embryos of *Picea asperata*. Its content increases continuously, functioning not only as a membrane structural component but also as a signaling molecule to activate downstream protein kinases, regulating embryo development and the expression of stress-resistant genes. No significant inhibition of lipid synthesis was observed throughout the process. Qu et al. [27] found that the drought-tolerant cultivar “Huajin” showed continuously enhanced lipid synthesis capacity under drought stress. The proportion of unsaturated fatty acids in membrane lipid components increased continuously, accompanied by the persistent accumulation of signaling lipids such as PA and PIP, which activated the expression of downstream drought-resistant genes. This showed a significant difference from the “first inhibition then promotion” pattern. This indicates that the response of lipid metabolism in woody plants to drought stress does not follow a single fixed pattern, but rather exhibits significant species specificity and environmental adaptive differentiation. The essence of such differences is closely related to the plants’ own drought tolerance potential and metabolic regulatory flexibility. The “first inhibition then promotion” pattern of lipid metabolism formed by *I. verticillata* in response to drought stress also provides an important theoretical basis for targeted breeding of drought-tolerant varieties.

## 5. Conclusions

*Ilex verticillata* exhibits distinct physiological response gradients and dynamically coupled characteristics with lipid metabolism under drought stress. In the early stage of drought, the relative water content of leaves decreases continuously, and chlorophyll synthesis is inhibited to reduce transpiration water consumption, while the activity of antioxidant enzymes increases to scavenge reactive oxygen species. Metabolomic analysis shows that 5 lipid and lipid-like metabolites among the top 30 differential metabolites are significantly down-regulated after 20 days of drought. It is speculated that the plant reduces the production of peroxidation substrates by inhibiting lipid synthesis to lower the risk of membrane lipid peroxidation damage, reflecting the synergy between the “drought avoidance” strategy and defensive metabolic inhibition. In the late stage of drought, the relative water content of leaves is significantly different from that of the control group, the activity of antioxidant enzymes continues to increase, and the content of MDA further intensifies, reflecting the progressive aggravation of membrane damage. At this time, lipid metabolism undergoes a transitional change, with all 17 lipid and lipid-like metabolites being up-regulated, including membrane lipid components such as phosphatidylcholine (PC) and phosphatidylglycerol (PG). By increasing membrane lipid saturation and accumulating membrane structural components, the stability and protective properties of cell membranes are enhanced, alleviating drought-induced membrane damage, thus forming an integrated response of “active defense” and compensatory metabolic activation.

Glycerophospholipid metabolism is a key pathway commonly enriched under both drought and rehydration conditions, and its dynamic changes run through the entire process of *I. verticillata*’s response to drought. In the early stage of drought, the contents of glycerophospholipids such as PC and phosphatidylethanolamine (PE) are down-regulated, reducing peroxidation-sensitive substrates to mitigate oxidative damage. In the late stage of drought, a large amount of membrane lipids such as PC and PG accumulate, remodeling membrane lipid composition to maintain membrane fluidity and integrity. After rehydration, 11 lipid metabolites remain up-regulated, and a small number are down-regulated, indicating that the glycerophospholipid metabolic pathway remains active. It accelerates the repair of the membrane system by stimulating lipid synthesis and membrane structure reconstruction, confirming the core regulatory role of this pathway in drought adaptation and rehydration recovery.

This study is the first to reveal the molecular basis of the “first inhibition then promotion” pattern of lipid metabolism in *I. verticillata*: that is, through metabolic inhibition in the early stage of drought to reduce energy consumption and oxidation risk, metabolic activation in the later stage to achieve membrane structure repair and function maintenance, and rapid reconstruction of membrane lipid homeostasis after rehydration, which reflects the phased adaptation strategy of *I. verticillata* to drought stress. As a core hub, the glycerophospholipid metabolic pathway, whose key metabolites (such as 1-acyl-sn-glycerol-3-phosphate glycerol and phosphatidylcholine) accumulate in a time-dependent manner and are closely related to the regulation of membrane stability, provides important targets for the breeding of drought-tolerant varieties. The research results enrich the theory of plant stress metabolic regulation and provide a typical example for analyzing the drought resistance mechanism of woody plants.

## Figures and Tables

**Table 1:** Comparative analysis of differential metabolites in *I. verticillata* during various drought phases.

Treatment Group	Total Identification Results of Metabolites	Total Number of Metabolites with Significant Differences	Total Number of Metabolites Significantly Up-Regulated	Total Number of Metabolites Significantly Down-Regulated
10 d vs. 10 dck	968	68	40	28
20 d vs. 20 dck	968	230	113	117
30 d vs. 30 dck	968	165	138	27
f1 vs. f1 ck	968	239	130	109

## Data Availability

The original contributions presented in this study are included in the article. Further inquiries can be directed to the corresponding author.
